# The level and factors associated with caregiver experience among parents of children with cerebral palsy: a cross-sectional study in Southwest China

**DOI:** 10.3389/fpubh.2025.1482011

**Published:** 2025-09-01

**Authors:** Xiaoying Zhong, Champa J. Wijesinghe

**Affiliations:** ^1^Department of Nursing, West China Second Hospital, Sichuan University/Key Laboratory of Birth Defects and Related Diseases of Women and Children (Sichuan University), Ministry of Education, Chengdu, Sichuan, China; ^2^Department of Community Medicine, Faculty of Medicine, University of Ruhuna, Matara, Sri Lanka

**Keywords:** cerebral palsy, child, caregiver experience, parents, depression, fatigue

## Abstract

**Background:**

Disability is a global public health issue, affecting one in seven people worldwide. In China, there are about 5 million children under 14 years of age with cerebral palsy and about 40,000 new individuals occur every year. Previous studies have revealed that the caregivers of children with cerebral palsy were more likely to perceive a greater burden compared with caregivers of typically developing children. However, there is a lack of information available on the care burden experienced by parents of children with cerebral palsy in China.

**Objectives:**

This study was conducted to determine the level of care burden and its related factors in the parents of children with cerebral palsy.

**Methods:**

This descriptive cross-sectional study was conducted with parents of 165 children with cerebral palsy who were enrolled in children rehabilitation departments of three tertiary hospitals between September 2021 and December 2022. Besides demographic information, the Caregiver Burden Inventory, the Patient Health Questionaire-9, and the Fatigue Severity Scale were used to collect data. Data were analyzed by descriptive and inferential statistics (correlation and multiple linear regression analysis).

**Results:**

The mean (±SD) Caregiver Burden Inventory score of the parents was 42.18 ± 18.79. The score of Fatigue Severity Scale and Patient Health Questionaire-9 demonstrated positive moderate to strong correlations with caregiver burden (*r* = 0.461, *p* < 0.001; *r* = 0.630, *p* < 0.001, respectively). The results of the multiple linear regression analysis showed that a low level of education, long caring time combined with visual impairment, higher depression, and fatigue had an influence on caregiver burden, and 46.4% of the variance in caregiver burden was explained by these factors.

**Discussion:**

The key predictors of caregiver burden include the level of education, caring time, children with visual impairment, and the degree of depression and fatigue. Efforts should be made to relieve the burden on parents of children with cerebral palsy.

## 1 Introduction

Disability is a global public health issue, affecting one in seven people worldwide (1). The World Health Organization reported that about 15% of the world’s population was living with disability in the year 2020, including about 93 million children and 720 million adults with significant difficulties in functioning (2). Developmental disorders in children encompass a range of conditions that affect a child’s physical, cognitive, or emotional growth and development (3). These disorders can impact various areas, including learning, communication, social skills, and daily functioning (3). Some common developmental disorders in children include Autism Spectrum Disorder (ASD), Down Syndrome, Attention-Deficit/Hyperactivity Disorder (ADHD), Dyslexia, Cerebral Palsy (CP), and so on (4–6). The World Health Organization (WHO) defines “Quality of Life” (QoL) as the individual’s perception of their position in life in the context of the culture and value systems in which they live and about their goals, expectations, standards, and concerns (7). Previous studies showed that caregivers of children with ASD and ADHD have a higher score of stigmas, burden, depression, anxiety, and a poorer QoL than normal (8, 9). Surveys revealed that the primary themes of stressful experiences among parents of young children with Down syndrome included emotional burdens, caregiving responsibilities, struggles against stigma and discrimination, concerns about the future, and challenges related to health, education, and financial issues (10–13). Approximately 95% of mothers with dyslexic children experience anxiety about their child’s future and academic performance, in addition to distress related to dyslexia (14–16). In China, among the mothers of 252 children with ASD, 26.02, 17.34, and 3.57% had mild, moderate, and severe depression, respectively (17). Caregivers of children with ASD scored significantly lower on adaptability than that of the norm (18), 46% of parents scored above the cut-off for stress (19), the total score of the Zarit caregiver burden Scale for caregivers of children with ASD was reported (52.62 ± 14.65) in Henan Province, China (20). A qualitative study conducted in China revealed that parents of children with ADHD use individual, family, and school coping strategies to cope with their stress (21). A meta-synthesis included eight qualitative types of research indicated that feeding pressures, educational concerns, language difficulties, and discrimination and stigmatization led to psychological, economic, and family stress in caregivers of children with Down syndrome (22). Four key categories of potential factors contributing to parental stress were identified: cultural factors, parents’ psychopathological symptoms, problem behaviors in children with developmental conditions, and caregiver burden (23–25).

Cerebral Palsy, with a rate of about 2.5 per 1,000 live births (26, 27). In China, there are about 5 million children under 14 years of age with cerebral palsy and about 40,000 new individuals occur every year (27, 28). Cerebral Palsy is a chronic illness in childhood, defined as a group of functional limitations that stem from challenges in the development of the central nervous systems, the continuous rehabilitation and lifelong support care are essential components of the development plan for a child with cerebral palsy (29).

Parenting a child with CP can be a unique journey filled with both challenges and rewards (30, 31). The key factors that contribute to positive experiences, include support networks, resilience, and personal growth, alongside those that lead to negative experiences, such as emotional and psychological stress, financial strain, isolation, and social withdrawal, navigating healthcare systems, concerns about the future, guilt and balancing family needs, fatigue and burnout, limited personal time, impact on family dynamics (32–35). The high medical expenses and education costs may turn into an economic burden for parents of children with cerebral palsy (28). The continued provision of excessive care leaves caregiver experiencing fatigue and depression, which can negatively impact their quality of life (28, 36, 37). Families may find it challenging to participate in social activities or events due to their child’s needs, leading to feelings of isolation or exclusion (38). While there are significant challenges, many parents also discover immense joy, growth, and fulfillment in their journey (39). The key often lies in seeking support, celebrating progress, and finding ways to cope with the difficulties that arise (40). Many parents report a profound emotional connection with their child with CP. The challenges they face together can strengthen their bond and create a deeper understanding of one another (41). Additionally, family members often become more compassionate and supportive, learning valuable life lessons about diversity and inclusion (42). Furthermore, this experience can lead to personal growth and a new perspective on life, prompting parents to develop patience, empathy, and problem-solving skills (43).

Caregiver burden refers to a multidimensional response to physical, psychological, emotional, social, and financial stressors associated with caring experience’ (44). Surveys conducted in Brazil (45), Portugal (46), and Turkey (36) have revealed that the caregivers of children with cerebral palsy were more likely to perceive a greater burden compared with caregivers of typically developing children. Within the context of the stress process model, numerous factors may affect the degree of burden for parents of children with cerebral palsy. Stigma is an important source of psychological stress for families of children with disability (47). Regarding caregiver characteristics, several studies show that having a lower income and education, living in a rural area, greater age, and poor health status are related to a higher burden in caregivers (36, 44, 48, 49). In terms of child characteristics, age, number of functional deficits, type of cerebral palsy, degree of disability, Gross Motor Function Classification System (GMFCS) level, and disease severity were associated with higher burden in caregivers (44, 48–51). The caregiver’s social support, self-efficacy, anxiety, depression, and fatigue were found to have significant correlations with caregiver burden (36, 45, 46, 48, 50, 52). A recent study reported the COVID-19 pandemic worsened the psychological status and the care burden of caregivers of children with cerebral palsy (53).

In pediatric psychology, examining the role of families can create an opportunity for understanding and treatment of chronic illness is well-documented (46). Bella et al. study has shown the level of burden and psychological maladjustment of parents has been linked to behavioral and emotional disturbance of children (45). Furthermore, screening parents for excessive burden and assisting them in finding appropriate coping mechanisms significantly contributed to the successful management of stressors and better social function in their children (42, 50, 54). In China, however, there is a lack of information available on the care burden experienced by parents of children with cerebral palsy. Therefore, the main aim of the current study was to determine the levels, correlates, and predictive factors of care burden among parents of children with cerebral palsy in China.

### 1.1 Research questions

*Q1.* What is the level of caregiver burden among parents of children with cerebral palsy in southwest China?

*Q2.* What demographic and socio-economic factors are associated with caregiver burden among these parents? a. How do parental age, education level, and employment status correlate with caregiver burden? b. Does the severity of the child’s condition correlate with the level of caregiver burden?

*Q3.* What depression and fatigue are associated with caregiver burden?

### 1.2 Hypotheses

*H1*: The level of caregiver burden among parents of children with cerebral palsy in southwest China is significantly higher than the general population, indicating a substantial impact on their daily lives.

*H2*: There is a significant positive correlation between the severity of the child’s cerebral palsy and the level of caregiver burden, such that parents of children with more severe disabilities report higher levels of burden.

*H3*: Lower levels of parental education and employment status are associated with higher caregiver burden, suggesting that socioeconomic factors play a crucial role in the experience of caregiving.

*H4*: Higher levels of depression and fatigue among parents are significantly associated with higher levels of caregiver burden.

## 2 Methods

### 2.1 Design

This cross-sectional study was performed in children’s rehabilitation departments of three tertiary hospitals in XX between September 2021 and December 2022. A local hospital’s Institutional Review Board approved this study.

### 2.2 Participants

This study was conducted among parents of children with cerebral palsy who were enrolled in children’s rehabilitation departments of three tertiary hospitals in Chengdu, Sichuan Province between September 2021 and December 2022. It was aimed that all parents who have a child with cerebral palsy would agree to participate in this study, without using sample selection. A total of 173 parents agreed to participate in the study, 8 parents were excluded because they provided insufficient/unreliable data, and 165 parents were finally included in the data analysis. The inclusion criteria of parents were as follows: (i) Caring for child aged 1–14 with cerebral palsy, (ii) parents who are taking on the responsibilities for the daily provision of care, (iii) living with the child, could read and write in Chinese. The inclusion criteria of children were as follows: (i) the children with cerebral palsy were diagnosed according to the diagnostic criteria of China Cerebral Palsy Rehabilitation Guidelines (2015), (ii) the children were aged 0–14 years old.

All participants self-completed the questionnaire in the presence of the principal investigator. The overall time taken to collect data by completing the questionnaires was on average 30–45 min.

### 2.3 Measures

Besides demographic information, the Caregiver Burden Inventory (CBI), the Patient Health Questionaire-9 (PHQ-9), and the Fatigue Severity Scale (FSS) were used to collect data. The selection of the CBI, PHQ-9, and FSS is pivotal for gaining insights into the experiences of caregivers, particularly parents of children with cerebral palsy. Each of these tools has been validated and widely used in research, providing crucial data to understand the intricacies of caregiver experiences. By employing these validated instruments, researchers and healthcare providers can identify specific needs and challenges, ultimately leading to improved support systems that enhance the well-being of both caregivers and their children. This comprehensive approach fosters a deeper understanding of the factors influencing caregiver burden, paving the way for effective interventions.

#### 2.3.1 Demographic characteristics of participants

The demographic characteristics questionnaire was composed of two parts. The first part included the basic information of parents, including the relationship to the child (father and mother), age, education level (primary school and below, junior high school, senior high school, university, master and above), occupation, marital status, average caring time (hours/day) and family systems (composed of nuclear family, stem family, joint family, single parent family). The definition of a nuclear family is a family unit that includes two married parents of opposite genders and their biological or adopted children living in the same residence (55). The stem family is composed of parents, unmarried children, and married sons and their wives (56). The joint family consists of parents and their unmarried children, two or more married sons, and their wives (56). The single-parent family consists of at least one dependent child and the mother or father, the other parent being dead or permanently absent (57).

The second part included the basic information about children with cerebral palsy, including age, gender, types of cerebral palsy, Gross Motor Function Classification System (GMFCS), visual impairment, and epilepsy. The types of cerebral palsy are comprised of Spastic hemiplegia, Spastic diplegia, Spastic tetraplegia, Ataxia, Dyskinetic, and Mixed types, according to China Cerebral Palsy Rehabilitation Guidelines (2015) (27). The gross motor skills (e.g., sitting and walking) of children and young people with cerebral palsy can be categorized into five different levels using a tool called the Gross Motor Function Classification System (58, 59).

#### 2.3.2 Caregiver Burden Inventory (CBI)

The Caregiver Burden Inventory (CBI) is a self-administered, multidimensional scale for assessing caregiver burden among caregivers of persons with dementia (60), children with cancer (61), psychiatric patients (62), and rheumatoid arthritis patients (63). This measure consists of 24 items divided into five domains: Time-dependence (1–5 items), developmental burden (6–10 items), physical burden (11–14 items), social burden (15–18 items), and emotional burden (19–24 items) (64). Each item is rated on a 5-point (0–4) Likert-type scale, with the total score ranging from 0 to 96 (65). The internal consistency of the *Chinese-*CBI was reported as 0.95 in China (64). In the present study, the Cronbach’s alpha of the CBI was 0.944, suggesting the internal consistency of the CBI was adequate. The CBI has been validated in various populations, showing strong correlations with other measures of psychological distress and quality of life (66). This established correlation makes it a reliable instrument for assessing caregiver experiences, particularly in parents of children with CP.

#### 2.3.3 Patient Health Questionaire-9 (PHQ-9)

The Patient Health Questionaire-9 (PHQ-9) is composed of nine items. It can be used to assess depressive symptoms of the general population over the last 2 weeks (67). The total score ranges from 0 to 27; a score of 10 or greater is defined as depression. In the Chinese validation study, the PHQ-9 was reported to have acceptable internal consistency (*α* = 0.90) (68). In the current study, the Cronbach’s alpha of the EPDS was 0.899, suggesting the internal consistency of the PHQ-9 was adequate. Research has shown that the PHQ-9 is strongly correlated with other measures of mental health and well-being (69). Its use in caregiving contexts has revealed significant associations between caregiver mental health and the burden they experience.

#### 2.3.4 Fatigue Severity Scale (FSS)

The Fatigue Severity Scale (FSS) is a self-administered questionnaire measuring fatigue (70). It includes nine statements rated on a 7-point scale. The total score is calculated as the mean of the responses to the nine statements (71). A score of 4 or higher indicates a moderate to high level of fatigue (72). In the Chinese validation study, the FSS was found to have a very good internal reliability (*α* = 0.93) (73). In this study, the Cronbach’s alpha of the FSS was 0.929. Previous studies have demonstrated a strong correlation between the FSS and other measures of health-related quality of life and psychological well-being (74, 75). It has proven particularly valuable in evaluating fatigue among caregivers, providing insights into how fatigue influences their overall burden and caregiving experiences.

### 2.4 Statistical analysis

Data were analyzed by SPSS 21.0 software using descriptive and inferential statistics. The sample size of this study was less than 200, so the normality test was judged using Kurtosis and Skewness, with Kurtosis ≤ 3 and Skewness ≤ 10 indicating that the data basically conformed to a normal distribution. Mean and standard deviation (SD) were reported for continuous variables that conformed to a normal distribution, and Median and interquartile spacing were reported for continuous variables that conformed to a non-normal distribution. Number and percentage were reported for categorical variables.

A stepwise model in multiple linear regression is a systematic method for selecting a subset of predictor variables to include in a regression model. This approach is particularly useful when dealing with numerous potential predictors, allowing researchers to identify the most significant ones while avoiding overfilling. The stepwise regression process can be conducted in several ways, including forward selection, backward elimination, and bidirectional elimination. (i) Start with no predictors in the model. (ii) Add the predictor that has the lowest *p*-value (indicating the strongest relationship with the dependent variable) to the model. (iii) Continue adding predictors one by one, each time selecting the one with the lowest *p*-value until no remaining predictors meet the entry criterion. Criteria for entry: A common criterion is a significance level (alpha) such as 0.05. If the *p*-value for a predictor is less than 0.05, it is added to the model.

In the current study, predictor variables include the relationship to the child, age, education level, occupation, marital status, average caring time and family systems, the score of the PHQ-9 and FSS, the response variable is the score of CBI, and the stepwise regression process was conducted by forward selection. The multiple linear regression was undertaken to evaluate the statistical significance of the effect of demographic factors, depression, and fatigue on caregiver burden by forward selection of the stepwise method. The statistically significant value was *p*-value <0.05. The assignment of predictor variables is in Table 1.

**Table 1:** The assignment of predictor variables.

Predictors variables	The assignment of variables
Age (years)	“≦30” = 1; “31–40” = 2; “≧41” = 3
Relationship to child	Father = 1; Mother = 2
Educational level	Primary school and below (≤6 years) = 1
	Junior high school (7–9 years) = 2
	Senior high school (10–12 years) = 3
	University (13–16 years) = 4
	Master and above (≥17 years) = 5
Occupation	Employed = 1; Unemployed = 2
Marital status	Married = 1; Divorced = 2
Average caring time (hours/day)	“<4” = 1; “4–8” = 2; “>8” = 3
Family systems	Nuclear family = 1; Stem family = 2; Joint family = 3; Single parent family = 4
Age of child (years)	“<3” = 1; “3–6” = 2; “>6” = 3
Sex of child	Boy = 1; Girl = 2
Types of Cerebral Palsy	Spastic hemiplegia = 1; Spastic diplegia = 2; Spastic tetraplegia = 3; Ataxia = 4; Dyskinetic = 5; Mixed types = 6
GMFCS	I level = 1; II level = 2; III level = 3; IV level = 4; V level = 5
Combined visual impairment	Yes = 1; No = 2
Concomitant epilepsy	Yes = 1; No = 2

GMFCS, Gross Motor Function Classification System.

## 3 Results

A total of 165 parents were included in this study. Regarding the age of participants, 32 (19.4%) were ≦30 years, 113 (68.5%) were 31–40 years, and 20 (12.1%) were ≧41 years. Most of the participants were child’s mothers (*n* = 129, 78.2%). Other demographic characteristics of participants are displayed in Table 2.

**Table 2:** Demographic characteristics of participants and the score of CBI (*n* = 165).

Variables	Number (*n*)	Percentage (%)	The score of CBI (Mean ± SD)	Kurtosis	Skewness
Age (years)					
≦30	32	19.4	42.78 ± 15.61	−0.874	0.057
31–40	113	68.5	41.20 ± 20.03	−0.914	−0.177
≧41	20	12.1	46.80 ± 15.99	−0.492	−0.053
Relationship to child					
Father	36	21.8	37.67 ± 19.60	−0.814	0.168
Mother	129	78.2	43.44 ± 18.43	−0.654	−0.286
Educational level					
Primary school and below (≤6 years)	10	6.1	58.50 ± 8.95	−2.200	−0.099
Junior high school (7–9 years)	22	13.3	42.55 ± 19.28	−0.831	0.357
Senior high school (10–12 years)	31	18.8	39.68 ± 20.13	−0.949	−0.380
University (13–16 years)	89	53.9	42.30 ± 17.93	−0.625	−0.161
Master and above (≥17 years)	13	7.9	34.15 ± 20.81	−0.634	0.439
Occupation					
Employed	75	45.5	46.04 ± 16.83	−0.771	−0.183
Unemployed	90	54.5	38.97 ± 19.80	−0.885	−0.073
Marital status					
Married	162	98.2	41.83 ± 18.70	−0.801	−0.189
Divorced	3	1.8	61.33 ± 15.37	0.000	1.658
Average caring time (hours/day)					
<4	35	21.2	36.25 ± 18.38	−0.638	0.021
4–8	34	20.6	31.94 ± 20.34	−0.803	0.336
>8	96	58.2	47.97 ± 16.13	−0.849	−0.197
Family systems					
Nuclear family	58	35.1	40.19 ± 18.27	−0.67	−0.236
Stem family	61	37	43.97 ± 18.80	−0.589	−0.289
Joint family	33	20	39.45 ± 19.46	−1.157	0.061
Single parent family	13	7.9	49.62 ± 18.58	−0.025	−0.39
Age of child (years)					
<3	82	49.7	42.45 ± 15.74	−0.118	0.078
3–6	54	32.7	42.54 ± 20.79	−1.215	−0.365
>6	29	17.6	40.76 ± 23.01	−1.211	−0.122
Sex of child					
Boy	99	60	43.90 ± 19.33	−0.816	−0.266
Girl	66	40	39.59 ± 17.75	−0.621	−0.140
Types of Cerebral Palsy					
Spastic hemiplegia	83	50.3	36.63 ± 19.00	−0.754	−0.186
Spastic diplegia	25	15.2	43.92 ± 16.80	−0.378	−0.500
Spastic tetraplegia	31	18.8	49.48 ± 15.53	−0.699	0.097
Ataxia	8	4.8	53.00 ± 19.00	0.000	0.000
Dyskinetic	2	1.2	49.00 ± 26.87	−1.536	−0.054
Mixed types	16	9.7	47.88 ± 18.61	−0.282	−0.968
GMFCS					
I level	70	42.4	36.44 ± 20.27	−0.940	0.155
II level	39	23.6	43.00 ± 17.10	−0.299	−0.154
III level	28	17	48.64 ± 15.78	−1.192	−0.088
IV level	14	8.5	43.64 ± 17.62	−0.419	−0.496
V level	14	8.5	54.21 ± 12.70	0.154	−0.262
Combined visual impairment					
Yes	69	41.8	47.23 ± 18.65	−0.357	−0.607
No	96	58.2	38.55 ± 18.13	−0.645	0.059
Concomitant epilepsy					
Yes	8	21.8	41.00 ± 15.50	0.423	−0.953
No	157	78.2	42.24 ± 18.98	−0.8	−0.181

CBI, Caregiver Burden Inventory; SD, Standard Deviation; GMFCS, Gross Motor Function Classification System.

The result indicated that the mean and SD of the CBI score was 42.18 ± 18.79. Among the five domains, time-dependent burden scored the highest (14.64 ± 4.91), followed by developmental burden (12.38 ± 5.78), while emotional burden scored the lowest (2.79 ± 3.36).

The scores of total CBI and domains were positively correlated with PHQ-9 and FSS (*r* ranging from 0.420 to 0.630 and 0.250 to 0.447, respectively, all *p* < 0.001). The mean and standard deviations of the variables under the study and their Spearman correlations are given in Table 3. The total scores of the PHQ-9, which measures levels of depression, exhibited a positive correlation with the scores from the Caregiver Burden Inventory. This indicates that higher levels of depressive symptoms in individuals, as assessed by the PHQ-9, are associated with higher caregiver burden. In other words, caregivers who reported more significant depressive symptoms also tended to experience greater feelings of burden related to their caregiving responsibilities.

**Table 3:** The mean and standard deviations of the variables under the study and their correlations (*n* = 165).

Variables	Mean	SD	1	2	3	4	5	6	7	8
1. PHQ-9	7.98	5.56	1							
2. FSS	4.21	1.25	0.513	1						
3. Time-dependent burden	14.64	4.91	0.420	0.250	1					
4. Developmental burden	12.38	5.78	0.533	0.428	0.705	1				
5. Physical burden	7.83	5.00	0.617	0.447	0.608	0.843	1			
6. Social burden	4.54	3.82	0.576	0.406	0.404	0.619	0.676	1		
7. Emotional burden	2.79	3.36	0.474	0.309	0.334	0.519	0.519	0.667	1	
8. Total burden of CBI	42.18	18.79	0.630	0.461	0.744	0.923	0.906	0.783	0.689	1

*p* < 0.001 for all correlations; SD, Standard Deviation; PHQ-9, Patient Health Questionaire-9; FSS, Fatigue Severity Scale; CBI, Caregiver Burden Inventory.

The total scores on the FSS, which assesses the severity of fatigue and its impact on daily functioning, exhibited a positive correlation with the scores from the CBI. This finding indicates that higher levels of fatigue reported by caregivers are associated with increased feelings of burden related to their caregiving roles. The positive correlation likely extends to specific domains of both the FSS and the CBI. This means that not only the overall fatigue levels but also particular aspects of fatigue (such as physical fatigue, mental fatigue, or the impact of fatigue on daily activities) may correlate with specific dimensions of caregiver burden (such as emotional, physical, or social burden). For instance, caregivers reporting significant physical fatigue may also experience heightened emotional or role-related burdens in their caregiving tasks.

The multiple linear regressions analysis revealed that having a low level of education, long caring time, having a child with combined visual impairment, and higher scores of PHQ-9 and FSS pertained to higher scores of CBI (adjusted *R*^2^ = 0.464, *F* = 29.357, *p* < 0.001). The results of the multiple linear regression analysis for the factors related to the caregiver burden of parents of children with cerebral palsy are presented in Table 4. Caregivers with a lower level of educational attainment were found to report higher levels of caregiver burden. This suggests that educational background may influence coping strategies and resources available to caregivers. The duration of caregiving is significantly linked to increased caregiver burden. Those who have been providing care for a longer period tend to experience greater levels of stress and exhaustion, likely due to the cumulative effects of long-term caregiving responsibilities. Caregivers of children who have combined visual impairment reported higher burden levels. This may reflect the unique challenges and demands associated with caring for a child with such impairments, which may require more intensive support and resources. Higher scores on PHQ-9 indicate greater depressive symptoms among caregivers, which are associated with increased feelings of burden. This suggests that as caregivers experience more depressive symptoms, their perceived burden also rises. Higher scores on the FSS were also significantly related to higher CBI scores, indicating that greater fatigue levels experienced by caregivers correlate with an increased sense of burden. This may be due to the physical and emotional exhaustion that caregiving can entail.

**Table 4:** The results of the multiple linear regressions analysis for the factors related to caregiver burden of parents of children with CP (*n* = 165).

Variables	B	SE	β	*t-*value	*p*-value
Constant	26.879	7.727	–	3.479	0.001
Education level	−2.231	1.121	−0.121	−1.991	0.048*
Average caring time (hours/day)	3.583	1.392	0.155	2.574	0.011*
Combined visual impairment	−6.133	2.214	−0.162	−2.77	0.006*
PHQ-9	1.618	0.23	0.479	7.029	<0.001**
FSS	2.68	1.016	0.179	2.638	0.009*

B, Unstandardized Coefficient; SE, Standard Error; β, Standardized Coefficient; *R*^2^ = 0.480, adjusted *R*^2^ = 0.464, *F* = 29.357, *, *p* ≤ 0.05, **, *p* ≤ 0.001.

## 4 Discussion

To the best of our knowledge, the current study presented the first quantitative research on caregiver burden related to parents of children with cerebral palsy in southwest China. This cross-sectional study was conducted to determine the level of care burden and its related factors in the parents of children with cerebral palsy. The finding of the study detected that the score of care burden in half of parents was of moderate level. In line with the studies conducted in South Africa (76), Spain (50), Turkey (36, 52), Nigeria (49), Poland (48), and Brazil (77), most of the parents of children with cerebral palsy endured moderate to severe care burden and various challenges on account of restricted access to alternative caring resources and the fear for children’s development. This high caregiver burden among parents of children with cerebral palsy calls for attention and action by healthcare institutions and disabled persons’ federations in China.

According to the result of this study, among of five domains of CBI, time-dependent burden received the highest score with a mean ± SD of 14.64 ± 4.9, followed by developmental burden scoring 12.38 ± 5.78 in comparison to other aspects. The heavy time-dependent burden can deplete the caregiver’s energy and increase negative emotions (78). The present study illustrated that parents of children with cerebral palsy tend to feel time-related pressure and restriction of their social and cultural activities due to multiple additional care tasks, which is consistent with previous studies (79, 80). The result of multiple linear regression analysis found that the level of education, caring time, visual impairment, the degree of depression, and fatigue remained statistically significant correlates of the caregiving burden.

According to a study conducted in Nigeria (77), parents of children with cerebral palsy who have higher education suffer less caring burden than those with low or no education. Barros et al. (77) pointed out that caregivers with lower educational levels are more likely to fall into poverty and undergo stress. Parents may face societal stigma regarding their child’s disability, which can be more pronounced for those who are less educated (66). This stigma can manifest as judgment or lack of understanding from others, leading to feelings of shame and isolation (37). Moreover, parents may internalize societal stigma, leading to feelings of worthlessness or failure in their parenting role (81). This can contribute to a negative self-image and increased caregiver burden (42). Addressing these issues requires targeted support, education, and community awareness to help alleviate the burdens faced by these families, fostering a more inclusive and understanding environment for parents and children with disabilities (82). By providing education and resources, communities can help reduce stigma and empower parents, ultimately improving their quality of life and the care they provide (83). Higher parental schooling, on the one hand, may enable them to search and acquire information and support resources more efficiently and on the other hand, foster self-efficacy in managing their children’s health issues and formulating effective plans to cope with their stressors (84). There is compelling evidence that education is one of the most important sources for caregivers to acquire competency, affecting the perception of stress, resilience, and psychological flexibility (85). For this reason, caregiver skill training was developed by the World Health Organization (WHO) aiming to facilitate access to parenting skills and strategies for caregivers of children with developmental delays or disabilities (86–89).

We found that the parents with longer caring time perceived a higher level of burden. This finding supports previous research that the length of caregiving is associated with caregiver burden. In terms of time cost, Park et al. (80) reported that parents of children with cerebral palsy spend about 14–15 h per day to support their children in Korea. A study conducted in Australia revealed a significant positive correlation between psychological problems and caring time (90). In the present study, the caring time on an average day was also fairly long, with 58.2% of parents spending more than 8 h per day devoted to caring for their children. On one hand, increased time allocated by the parents for their children might require one of the parents to quit work to care for the child, which adds a financial burden to the families. On the other hand, parents with longer caring time might feel more burnout and pain, which, in turn, enhances the care burden experienced as well (36).

The results of the current study demonstrated that the caregiver burden was higher in parents of children with visual impairment, which is consistent with previous research (44, 91). This might be attributed to the fact that visually impaired children need additional care for daily tasks due to their diminished independent living skills and increased likelihood of injury (44). Most of the qualitative studies conducted with caregivers of children with cerebral palsy documented that most caregivers worry about their children’s future (42). Thus, parents of visually impaired children with cerebral palsy are more likely to feel tiredness and pressure.

In keeping with the views in the literature (48, 92), it was observed that caregiving burden was significantly affected by depression and fatigue. Caregivers experiencing higher levels of depression may feel more overwhelmed by their responsibilities, which can create a cycle of increased stress and emotional strain (93). This highlights the importance of addressing the mental health needs of caregivers, as their psychological well-being is closely linked to their experience of burden (94). This has major clinical implications for medical practitioners and nurses in being able to identify parents at risk for burden. The current study suggests a significant relationship between the experience of fatigue and the perception of caregiver burden. Caregivers who report higher levels of fatigue may feel less capable or more drained in their caregiving roles, contributing to a greater sense of burden (95). This can create a vicious cycle where fatigue exacerbates feelings of burden, potentially leading to further fatigue and emotional distress (96). This highlights the necessity for interventions and support systems aimed at addressing fatigue among caregivers, which could, in turn, alleviate their burden and improve their overall quality of life (97). Parents with a greater number of depressive and fatigue symptoms may require more individualized support and interventions, such as problem-solving therapy and group educational intervention (98, 99).

The study contributes to providing existing evidence on caregiver burden in a Chinese context. However, there are a few limitations of this study. Firstly, the nature of cross-sectional study prevents the inference of causal relationships. Secondly, the overall multiple regression models explained only up to 46.4% of the variance in caregiver burden, which was relatively low, indicating the need for more extensive research on the correlates of caregiver burden. Third, limitation of quantitative approaches, particularly regarding the inability to capture the nuanced experiences and specific emotions that parents may face. While our quantitative design allowed for the measurement of broader trends and relationships, it did not provide the depth of understanding that qualitative methods offer.

## 5 Conclusion

In conclusion, this study showed that most parents of children with cerebral palsy experience moderate to severe caregiver burden, the time-dependent burden being the heaviest compared with other types of burden. A moderate burden may refer to caregivers who feel overwhelmed but can still manage their responsibilities, while a severe burden indicates that they are experiencing significant distress and may struggle to cope with their caregiving duties. The key predictors of caregiver burden include the level of education, caring time, children with visual impairment, and the degree of depression and fatigue. These findings underscore the multifaceted nature of caregiver stress and the importance of addressing these factors to support caregivers effectively. The current findings may prove useful for providing more effective family-oriented support programs for parents of children with cerebral palsy. Taking into account the factors linked to caregiver burden, further studies should examine the burden of care in different societies with diverse cultures.

## Data Availability

The original contributions presented in the study are included in the article/supplementary material, further inquiries can be directed to the corresponding author.
